# Detecting Breast Cancer with a Dual-Modality Device

**DOI:** 10.3390/diagnostics7010017

**Published:** 2017-03-18

**Authors:** Kamila Padia, Tania S. Douglas, Lydia L. Cairncross, Roland V. Baasch, Christopher L. Vaughan

**Affiliations:** 1 Department of Radiology, 2 Military Hospital, Hospital Street, Wynberg 7800, South Africa; kamilanaidoo@iafrica.com; 2 Medical Imaging Research Unit, University of Cape Town, Observatory 7925, South Africa; tania.douglas@uct.ac.za; 3 CapeRay Medical (Pty) Ltd, 51 Bell Crescent, Westlake Business Park 7945, South Africa; roland@caperay.com; 4 Department of Surgery, Groote Schuur Hospital and University of Cape Town, Observatory 7925, South Africa; lydia.cairncross@uct.ac.za

**Keywords:** breast cancer, dense breasts, dual-modality imaging, full-field digital mammography (FFDM), automated breast ultrasound (ABUS)

## Abstract

Although mammography has been the gold standard for the early detection of breast cancer, if a woman has dense breast tissue, a false negative diagnosis may occur. Breast ultrasound, whether hand-held or automated, is a useful adjunct to mammography but adds extra time and cost. The primary aim was to demonstrate that our second-generation Aceso system, which combines full-field digital mammography (FFDM) and automated breast ultrasound (ABUS) in a single platform, is able to produce improved quality images that provide clinically meaningful results. Aceso was first tested using two industry standards: a Contrast Detail Mammography (CDMAM) phantom to assess the FFDM images, and the CIRS 054GS phantom to evaluate the ABUS images. In addition, 25 women participated in a clinical trial: 14 were healthy volunteers, while 11 were patients referred by the breast clinic at Groote Schuur Hospital. The CDMAM phantom results showed the FFDM results were better than the European Reference (EUREF) standard of “acceptable” and were approaching “achievable”. The ABUS results showed a lateral and axial spatial resolution of 0.5 mm and an adequate depth penetration of 80 mm. Our second-generation Aceso system, with its improved quality of clinical FFDM and ABUS images, has demonstrated its potential for the early detection of breast cancer in a busy clinic.

## 1. Introduction

For the past 50 years, mammography—X-ray images of cranio-caudal (CC) and medio-lateral oblique (MLO) views of both breasts—has been the gold standard to diagnose healthy women for the early detection of breast cancer [1]. Although there is evidence that mammography has reduced the mortality rate among screened populations [2], some recent reports have suggested otherwise [3]. The sensitivity of full-field digital mammography (FFDM) varies from 75% to 90%, while the specificity ranges from 90% to 95% [4]. It has long been recognized that mammography performs poorly if a woman has dense breast tissue, which is often the case for pre-menopausal women younger than 50, and the sensitivity falls to less than 50% [5]. The dense fibro-glandular tissue masks the underlying tumours and a false negative diagnosis can have devastating consequences for the patient: a poorer prognosis and more expensive treatment [6].

Ultrasound is an imaging modality that, while lacking the spatial resolution of X-rays, is able to distinguish different tissue densities remarkably well, and does not suffer the disadvantage of ionizing radiation [7]. Hand-held ultrasound (HHUS) has been employed as an adjunct to X-ray mammography for over 50 years and plays a key role in the diagnosis of breast cancer in both younger and older women and during the subsequent biopsy procedure [8]. Large cohort studies over the past decade have shown that combined screening with FFDM followed by HHUS versus FFDM alone, especially in women with dense breast tissue, has resulted in increased sensitivity but a slight reduction in specificity [9,10,11]. Tagliafico et al. [12] compared adjunctive screening using either digital breast tomosynthesis (DBT) or HHUS in women with dense breasts, and established that the incremental cancer detection rate for DBT was 4.0 per 1000 women screened, while the rate for HHUS was 7.1 per 1000 screens. Since many breast clinics are now considering an upgrade from FFDM to DBT, this is an important finding because it suggests that ultrasound appears to be a better choice than tomosynthesis for adjunctive screening [13].

Since HHUS can be time-consuming and suffers from repeatability problems [14], automated breast ultrasound (ABUS) devices, where the patient lies on a bed—either in a supine position and her breasts are compressed under gravity [15] or in a prone position [16]—have been developed [17] and tested [18]. With these two types of ABUS design, three-dimensional (3D) volumetric information is acquired, either by a linear B-mode ultrasound probe that scans across the breast in the frontal plane [15] or by a ring transducer immersed in water that moves upwards from the nipple to the chest wall [16]. Strong evidence to support the use of FFDM followed by ABUS, particularly in women with mammographically dense breasts, was recently published by Giuliano and Giuliano [19], who showed that the addition of ABUS resulted in the detection of 12.3 breast cancers per 1000 women screened compared to 4.6 per 1000 by FFDM alone. Brem et al. [20] showed that combined FFDM plus ABUS produced an additional 1.9 detected cancers per 1000 screened but also led to an increase in the number of false positive findings.

Recognising that a single system designed to acquire both FFDM and ABUS images simultaneously could be advantageous for breast screening, we first proposed the development of a dual-modality device [21] and then built and clinically tested such a system [22]. The primary aim of the current paper is to demonstrate that our second-generation system is able to produce improved quality X-ray and ultrasound images that provide clinically meaningful insights.

## 2. Materials and Methods

### 2.1. Dual-Modality Aceso System

**Figure 1:**
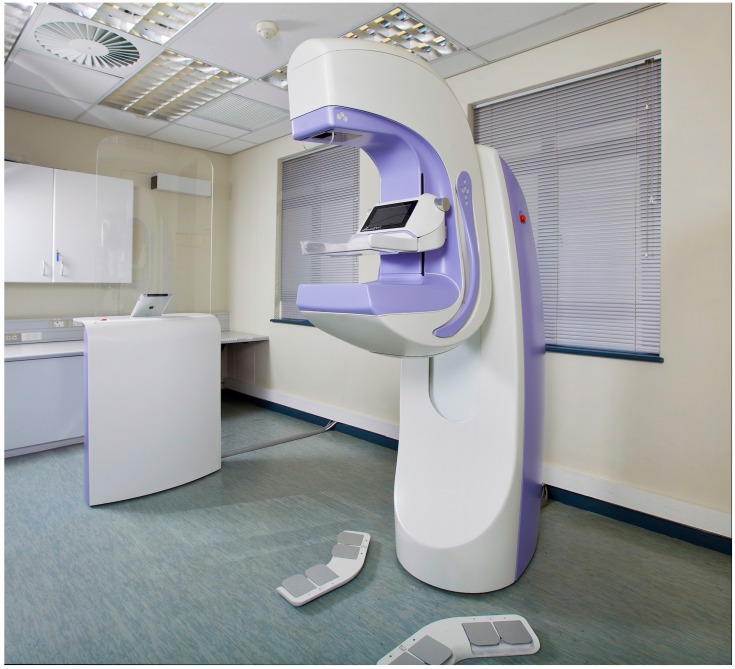
The Aceso dual-modality system as installed for the clinical trial, showing the acquisition workstation with iPad, a pair of foot pedals, gantry, and C-arm.

The first generation of our Aceso system—named after the Greek goddess of healing—implemented FFDM using a slot-scanning approach, while ABUS was accomplished by positioning a linear ultrasound transducer parallel to the X-ray camera [22]. Both the FFDM camera and the ABUS transducer moved from right to left and were located underneath the breast in a hermetically sealed platform. Our second-generation Aceso system utilized in the current study has the same basic geometry (Figure 1) but has incorporated a custom-designed linear ultrasound transducer manufactured by Vermon (180 Rue du Général Renault, Tours, France) that is 192 mm in length, with an element pitch of 0.5 mm (i.e., there are 384 elements), and a centre frequency of 6.5 MHz. The value chosen for centre frequency was a trade-off between depth of penetration (low value) and spatial resolution (high value). In addition, a new 128-channel beam former, with a built-in 3:1 multiplexer, manufactured by Telemed (Dariaus ir Gireno str. 42, Vilnius, Lithuania), drives the 384 transducer elements. The X-ray camera now moves at a slower rate of just 14 mm/s, thereby increasing the exposure time and improving the quality of the FFDM images [23].

A single FFDM image of the breast, gathered in the horizontal plane for the cranio-caudal view, has a field of view of 220.2 mm × 227.6 mm and is recorded as a 16-bit gray scale image of 4078 × 4214 pixels. The 235 ABUS images, gathered in the sagittal plane at 1 mm intervals, have a field of view of 162.8 mm × 81.4 mm and are recorded as 8-bit gray scale images of 1024 × 512 pixels. The standard Digital Imaging and Communication in Medicine (DICOM) file format is used to record the images, while the necessary header information in the files enables the FFDM and ABUS images to be co-registered based on the same coordinate system [22].

### 2.2. Phantom Testing

Prior to our clinical testing, we used two industry standard phantoms to evaluate our second-generation Aceso system: a Contrast Detail Mammography (CDMAM) phantom, manufactured by Artinis Medical Systems (Einsteinweg 17, Elst, The Netherlands); and a Model 054GS ultrasound phantom, manufactured by CIRS (2428 Almeda Avenue, Norfolk, VA, USA). CDMAM is the accepted European standard for evaluating FFDM image quality [24] and utilizes freely available software to automatically process images [25]. Zerdine, which simulates the acoustic properties of breast tissue, is used in the construction of the CIRS phantom, which also includes a variety of targets that mimic breast lesions.

### 2.3. Human Subjects

Fourteen healthy volunteers were recruited via advertisements placed at strategically visible points in Groote Schuur Hospital (GSH). In addition, 11 patients—of whom 9 had biopsy-proven breast cancer—were recruited through the GSH breast clinic by one of us (L.L.C.). The patients were evaluated before surgery and other treatment commenced. Our clinical protocol was approved by the Human Research Ethics Committee of the Faculty of Health Sciences at the University of Cape Town (project code 580/2013, 11 May 2015). Prior to their participation in the study, all subjects signed an informed consent form. Volunteers, who had no previous history of breast disease, were aged between 45 and 60, while patients ranged from 38 to 66 years. A set of four dual-modality images (FFDM and ABUS) were gathered for each subject: CC and MLO views for the left and right breasts.

For each of the 25 subjects, our radiologist (K.P.) compared the quality of the Aceso FFDM images with a predicate device—Hologic Selenium—used in her own practice at the Number 2 Military Hospital in Cape Town. It should be noted that she did not have the benefit of images acquired for each subject by her Selenium system to facilitate side-by-side comparison. Based on the Food and Drug Administration (FDA) guidelines [26], 12 parameters were compared (Table 1). One of three scores was given for each parameter: −1 if Aceso was judged by the radiologist to be worse than the predicate device; 0 if the two devices were deemed equivalent; and +1 if Aceso was considered better than the predicate device. Breast density was scored using the BI-RADS scale: A = almost entirely fatty; B = scattered fibro-glandular densities; C = heterogeneously dense; and D = extremely dense. Lastly, we recorded the time that each subject spent in the room for image acquisition.

## 3. Results

### 3.1. Phantom Testing

**Figure 2:**
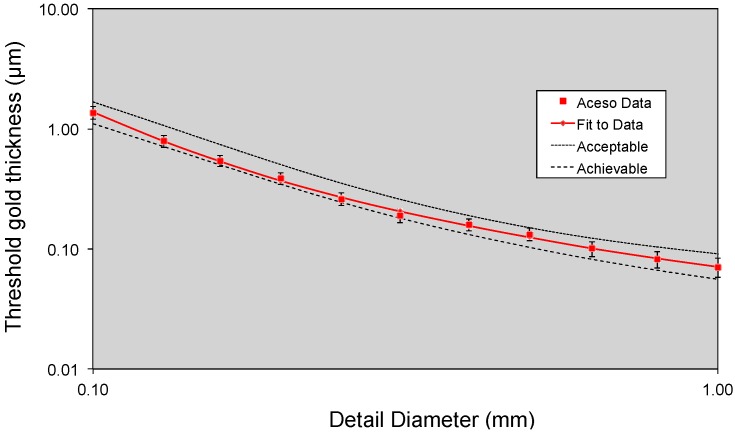
Data for the Contrast Detail Mammography (CDMAM) phantom generated by the European Reference (EUREF) software package [25]. The threshold gold thickness has been plotted as a function of detail diameter, using a logarithmic scale for both axes. The Aceso data are based on eight sequential X-ray images (at 33 kV, 30 mAs, 2.4 mGy) and may be compared with the EUREF standards of “acceptable” and “achievable” [24].

Our Contrast Detail Mammography (CDMAM) phantom (serial number 1809, Version 3.4) was located between two 20-mm-thick PMMA plates and placed on the breast platform, thus providing a total attenuation equivalent to 60 mm of breast tissue. The X-ray tube, using a W/Al target/filter combination, used values of 33 kV and 30 mAs, which were based on automatic exposure control values for 50 mm of PMMA. Using the method of Dance et al. [27], a mean glandular dose (MGD) of 2.4 MGy was estimated. The European Reference (EUREF) software package [25] required eight consecutive images of the CDMAM phantom to be entered, while the resulting Figure 2 illustrates the system’s performance. Note that each axis uses a logarithmic scale, while the threshold thicknesses of the gold disks embedded in the phantom are plotted against the disk diameters. The resulting curve for Aceso may be contrasted with the EUREF standards of “achievable” and “acceptable” [24].

**Figure 3:**
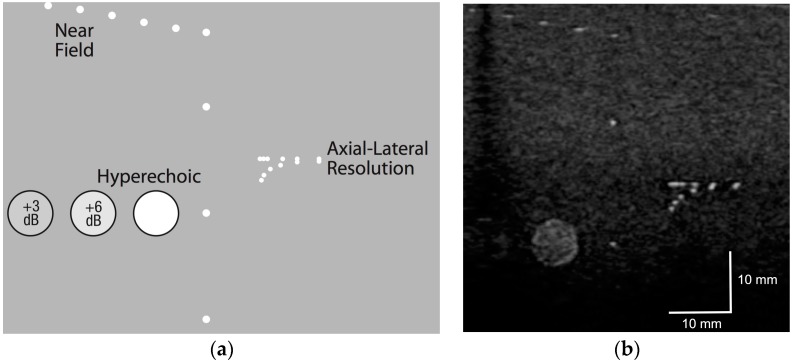
The CIRS 054GS ultrasound phantom: (**a**) template showing the various targets; and (**b**) data captured by the ultrasound transducer prior to the start of the clinical trial.

We placed the CIRS 054GS phantom on top of a 2-mm-thick Zerdine sheet on the breast platform and the ultrasound transducer was scanned from right to left. A template for the near field, axial-lateral resolution and hypoechoic targets may be seen in Figure 3a, and a single slice generated by our ABUS system is illustrated in Figure 3b. Based on these two images, the spatial resolution in the lateral direction was 0.5 mm, while the axial resolution was also 0.5 mm. In the scanning direction, the resolution was 1 mm, while the depth penetration in the axial direction was 80 mm.

### 3.2. All Human Subjects

The average age for all our subjects was 51.1 years, and the mean time taken by the radiographer with each subject to acquire a full set of images was 10 min and 6 s, while the average values for the 12 FFDM parameters as assessed by our radiologist (K.P.) are presented in Table 2. The BI-RADS breast density data for the 25 subjects are as follows: A = 5; B = 14; C = 4; D = 2.

### 3.3. Two Clinical Examples

Patient 1 was a 47-year-old woman who presented with a one-week history of a painless left breast lump. She had a medical history of a right-sided breast abscess treated five years previously and a left breast cyst aspirated 15 years earlier. She was a low-risk patient with no family history, had a child born in her twenties, and no previous use of systemic hormonal therapy. Clinical examination revealed a large, smooth, mobile left breast mass in the 3 o’clock position. There were no associated skin changes or lymphadenopathy.

**Figure 4:**
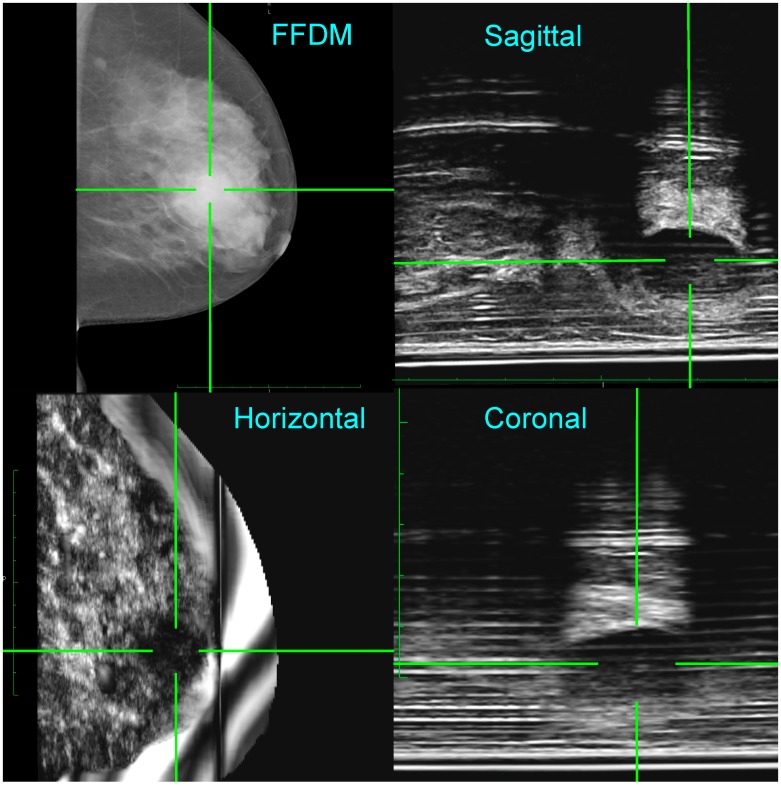
Co-registration of the FFDM in the horizontal plane and the ABUS images in the horizontal, coronal, and sagittal planes for Patient 1. A lesion (benign cyst) has been highlighted by cross hairs in both the FFDM and ABUS views. Note that for the ABUS images, the sagittal plane view is the acquired image, whereas the coronal and horizontal plane views have been reconstructed.

Figure 4 (top left) shows an FFDM image for the left medio-lateral oblique (LMLO) view, where the radiologist (K.P.) identified a dense area behind the nipple, highlighted by the green cross hairs, with two smaller opacities in the left upper quadrant. The 235 ABUS images were acquired simultaneously to the FFDM image in the sagittal plane and may be viewed as a video clip. Because the location of the ultrasound probe in the scan direction is known, 3D reconstruction of the ABUS data was performed. As seen in Figure 4, the three orthogonal ultrasound views confirm a 2.3 cm cyst—suggested by the acoustic enhancement—in the retro-areolar area. Note that the 3D location of the lesion is clearly identified by the co-registered green cross hairs in the FFDM and ABUS images. Following needle biopsy, cytology, and histology were benign and consistent with a breast cyst and some areas of acute inflammation. Her lump resolved after aspiration.

Patient 2 was a 61-year-old woman who presented with a four-week history of a painless left breast lump. She had no family history of breast cancer, had four children, and had not used hormone replacement therapy. On clinical examination, a 2 cm suspicious, hard, irregular breast mass was palpated with no associated lymphadenopathy.

**Figure 5:**
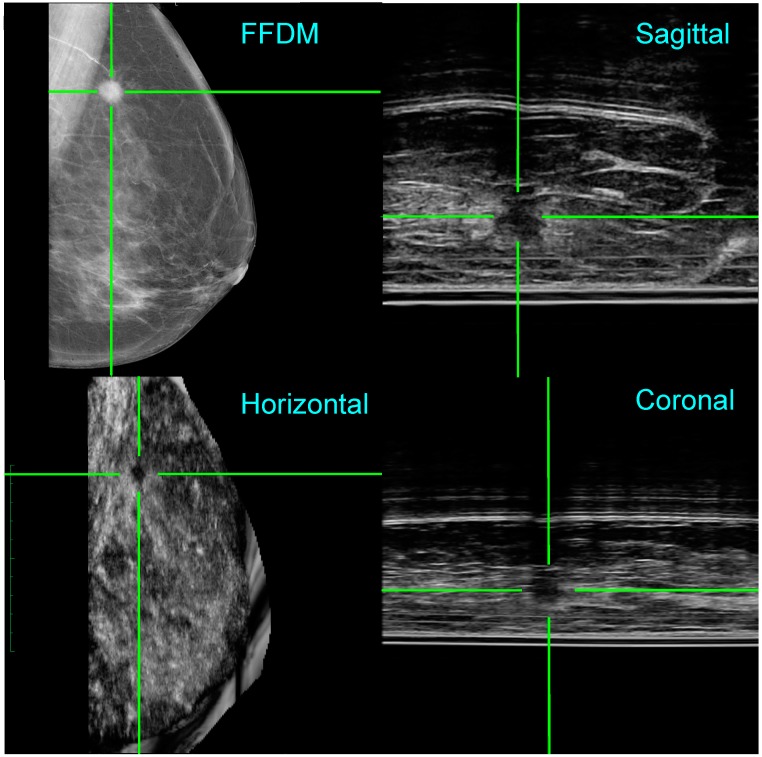
Co-registration of the FFDM in the horizontal plane and the ABUS images in the horizontal, coronal, and sagittal planes for Patient 2. A malignant lesion has been highlighted by cross hairs in the ABUS views and is clearly co-registered in the FFDM image. Note that, for the ABUS images, the sagittal plane view is the acquired image, whereas the coronal and horizontal plane views have been reconstructed.

Figure 5 (top left) shows an FFDM image for the left medio-lateral oblique (LMLO) view, where the radiologist (K.P.) identified a spiculated lesion in the outer quadrant (highlighted by the green cross hairs). The video clip of the ABUS images acquired in the sagittal plane at the same time as the FFDM image, illustrated the brief appearance of an irregularly shaped lesion located mid-way between the breast platform and the upper surface of the breast. As seen in Figure 5, the four views illustrate the co-registration of the FFDM and ABUS images generated by Aceso, with the 3D location of the lesion clearly identified. Following needle biopsy, cytology and histology confirmed an invasive ductal carcinoma. The patient underwent breast conserving surgery (by L.C.) and an axillary node clearance followed by adjuvant radiotherapy and hormonal therapy.

## 4. Discussion

The primary purpose of this study was to demonstrate that our second-generation Aceso system was able to produce FFDM and ABUS images that provided clinically meaningful insights. In particular, we were eager to demonstrate improved quality images compared to our first generation system [22]. All testing was conducted at Groote Schuur Hospital and took place over a period of five weeks, with a maximum of three women seen on a single day. The average acquisition time was only 10 min, and was marginally less (by just one minute) than our previous study, and comparable to the time required for FFDM-only systems, where times varied between 8 and 11 min [28].

The quality of the FFDM images may be assessed from Figure 2, Table 2, and Figure 4 and Figure 5. As seen in Figure 2, the curve for Aceso is better than the “acceptable” curve published by EUREF and approaches their “achievable” curve [25]. In Table 2, there are two parameters—breast positioning in the medio-lateral oblique view, and exposure of the breast tissue underlying the pectoralis muscle—where 40% of the Aceso images were judged by the radiologist (K.P.) to be inferior to the predicate device. These two parameters relate directly to the width of the breast platform in the medio-lateral direction, which, at 220 mm, was inadequate for women with large breasts. This can also be seen in Figure 5 for Patient 2. Aside from the inadequate coverage, the quality of the FFDM images in Figure 4 and Figure 5 was judged (by K.P.) to be diagnostically equivalent to her predicate device.

The quality of the ABUS images may be gauged from Figure 3, Figure 4 and Figure 5. When the phantom data of Figure 3b are compared with the template in Figure 3a, Aceso has good spatial resolution of 0.5 mm in the lateral and axial directions. In addition, because the transducer in our second-generation Aceso system is 192 mm long, it covers the region from the chest wall to beyond the nipple, thus gathering sagittal plane images of the largest breasts with just a single scan. The improved spatial resolution is particularly evident in Figure 5 (top right), where the connective tissue structures can be clearly seen in the sagittal plane ABUS image. However, image quality is directly affected by the acoustic coupling between the breast and the platform made from polymethylpentene, commonly called TPX, which is especially evident in Figure 4. Although there are relatively few artifacts in the acquired sagittal plane—horizontal lines parallel to the platform—these artifacts become particularly evident in the reconstructed coronal plane. Better coupling, as seen in Figure 5, will reduce the artifacts and enable our Aceso system to produce images of comparable quality to existing ABUS systems [15,20].

To address the “peripheral volume” problem—where the tissue on the periphery of the breast, particularly underneath the nipple, is not in contact with the TPX platform—we developed a custom breast pad that was tested on a number of subjects. Our results were unfortunately mixed, suggesting that further work needs to be done with this approach. Note that, for the two clinical examples seen in Figure 4 and Figure 5, the breast was coupled directly to the platform with ultrasound gel. This suggests that, until we have solved the peripheral volume problem, our Aceso system will be best suited to screening women with dense breast tissue.

Mammographic evaluation of patients with breast symptoms often requires the addition of ultrasound to establish an accurate BI-RADS score. In some clinics, it is standard practice for patients with detected lumps to be examined with HHUS so that, if necessary, a biopsy can be performed immediately. However, in high volume breast clinics, mammographic reporting may only take place after patients have left the clinic, making recall for ultrasound necessary with associated inconvenience and increased health care costs.

Ideally, clinical assessment should place the patient at the centre and ensure that all required examinations are performed from the start. A dual-modality device such as Aceso would significantly reduce the number of recalls due to simple cysts because the ultrasound and the mammogram would be done simultaneously, enabling the discovery of cancers that are not seen on FFDM but are visible with ABUS. We believe that the potential impact on clinical services of such a device may be significant, particularly within the public health sector where radiology consultant support for immediate reporting and hand-held ultrasound may be limited.

## 5. Conclusions

Even though FFDM is still regarded as the gold standard for the early detection of breast cancer, it is also recognised that, if a woman has dense breast tissue, lesions may be mammographically occult. The false negative findings can be devastating for the women concerned because a later diagnosis may lead to a poor prognosis and more expensive treatment. We believe that our second-generation Aceso system, with its improved quality FFDM and ABUS images, has demonstrated its potential for the early detection of breast cancer in busy clinics.

## Figures and Tables

**Table 1:** Parameters recommended by the Food and Drug Administration (FDA) to judge the quality of full-field digital mammography (FFDM) images [26].

Parameter	Definition
Breast positioning	Assess coverage of the breast on cranio-caudal (CC) and medio-lateral oblique (MLO) views
Exposure	Assess visualization of the adipose and fibroglandular tissues and visualization of breast tissue underlying the pectoralis muscle
Breast compression	Assess overlapping breast structures, uniformity of exposure of fibroglandular tissues, adequacy of penetration of thicker portions, exposure of thinner areas, and motion unsharpness
Image contrast	Assess differentiation of subtle tissue density differences
Sharpness	Assess the edges of fine linear structures, tissue borders, and benign calcifications
Tissue visibility	Assess the tissue visibility on the skin line
Noise	Assess noise obscuring breast structures or suggestive of structures not actually present
Artifacts	Assess artifacts due to image processing, detector failure, and other factors external to the breast
Image quality	Assess the overall clinical image quality

**Table 2:** Mean values for the 25 subjects (14 healthy volunteers and 11 patients), including age, the time taken by the radiographer with each subject in the image acquisition room, and the 12 FFDM parameters as assessed by our radiologist (K.P.) according to the FDA guidelines [26].

Parameter		Mean Value
Breast positioning	Cranio-caudal	–0.16
	Medio-lateral oblique	–0.40
Exposure	Adipose	0.00
	Fibroglandular	0.00
	Pectoralis	–0.40
Breast compression		0.00
Image contrast		–0.12
Sharpness		–0.12
Tissue visibility		–0.08
Noise		0.00
Artifacts		–0.24
Image quality		–0.08
